# A Case of a Rapidly Infected Giant Pulmonary Cyst Complicated by Abscess Formation: Treatment and Management

**DOI:** 10.7759/cureus.91500

**Published:** 2025-09-02

**Authors:** Eitetsu Koh

**Affiliations:** 1 Department of Thoracic Surgery, Tokyo Women's Medical University Yachiyo Medical Center, Yachiyo, JPN

**Keywords:** lobectomy, lung abscess, pulmonary cyst, streptococcus anginosus, thoracic surgery

## Abstract

Pulmonary cysts are air- or fluid-filled parenchymal spaces surrounded by an epithelial or fibrous wall; diagnosing an infected cyst can be challenging. Herein, we report a case of a 44-year-old woman who presented with fever and a productive cough. Chest computed tomography revealed a giant cystic lesion in the left upper lobe with an air-fluid level, suggesting superimposed infection. Despite intravenous antibiotics, the lesion rapidly progressed, leading to pulmonary abscess formation. A lobectomy was performed due to poor response to medical therapy and risk of sepsis. Pathology confirmed a giant pulmonary cyst with secondary abscess. This case illustrates the rare but serious complication of cyst infection leading to lobectomy and emphasizes the importance of early intervention and surgical consideration.

## Introduction

Pulmonary cysts are air- or fluid-filled parenchymal spaces surrounded by an epithelial or fibrous wall. They may be congenital (e.g., congenital pulmonary airway malformation, or CPAM), acquired (e.g., emphysematous bullae), infectious (e.g., post-tuberculosis or parasitic), or neoplastic. Most pulmonary cysts remain stable and asymptomatic; however, recognized complications include rupture, pneumothorax, hemoptysis, and secondary infection, while progression to abscess is uncommon in immunocompetent patients [1]. On high-resolution CT, diffuse cystic lung diseases demonstrate characteristic patterns that aid in differentiation from other cavitary processes [2].

Diagnosing an infected cyst may be challenging due to its overlap with necrotizing pneumonia, cavitary neoplasms, or parasitic etiologies such as echinococcosis [3-5]. The decision to pursue surgical management depends on symptom progression, treatment response, and radiologic findings. We present a case of a giant pulmonary cyst that evolved into a frank abscess despite antimicrobial therapy, highlighting the role of early surgical intervention.

## Case presentation

A 44-year-old woman with no significant medical or travel history presented with a five-day history of productive cough and low-grade fever. She denied dyspnea, hemoptysis, chest pain, or weight loss. Physical examination revealed diminished breath sounds over the left upper chest. Laboratory tests showed leukocytosis (13,200/μL), elevated C-reactive protein level (13.6 mg/dL), and normal liver and renal function.

**Figure 1:**
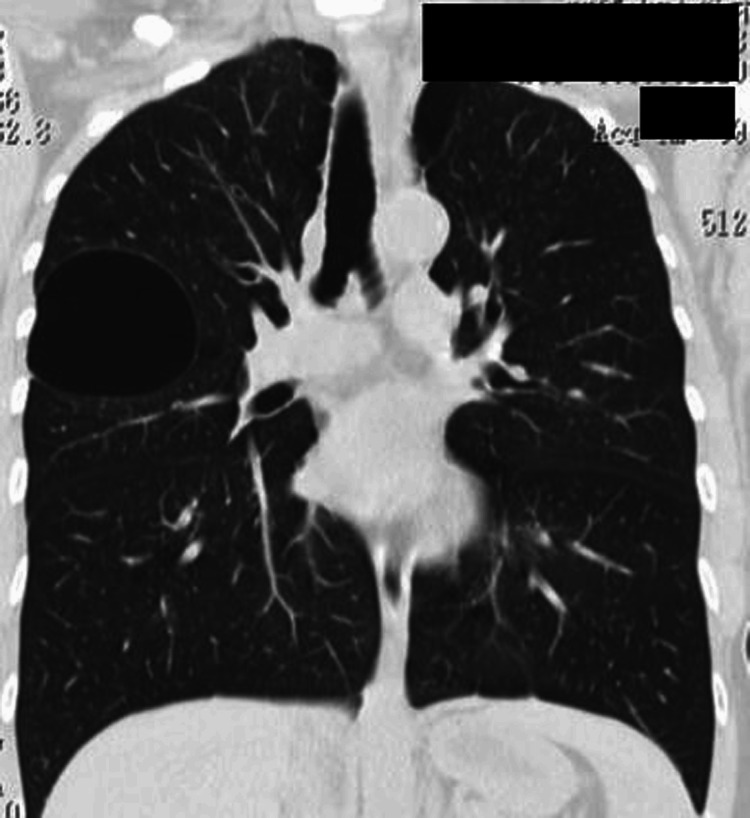
Coronal CT on initial presentation An air-filled cyst (9 cm) is seen in the apicoposterior segment of the right upper lobe with no fluid or consolidation.

Her initial chest radiograph (day 0) was inconclusive. Contrast-enhanced chest CT revealed a well-defined, 9-cm air-filled cyst in the right upper lobe apicoposterior segment, without consolidation or fluid (Figure 1). Differential diagnoses included a congenital cyst, emphysematous bulla, or a parasitic cyst (hydatid), though serologic testing for Echinococcus was negative.

Empirical intravenous meropenem was initiated at 1 g every eight hours (approx. 48 mg/kg/day) based on body weight (60 kg). Bronchoscopy was deferred due to the risk of cyst rupture and stable oxygenation.

**Figure 2:**
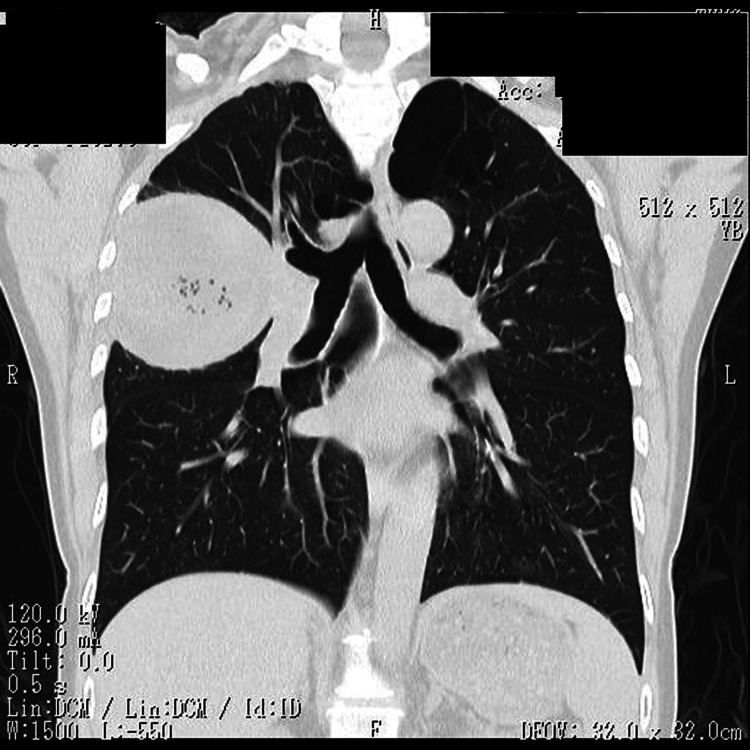
Chest CT at the time of clinical deterioration Development of an air-fluid level within the right upper lobe cyst, suggesting abscess formation.

**Figure 3:**
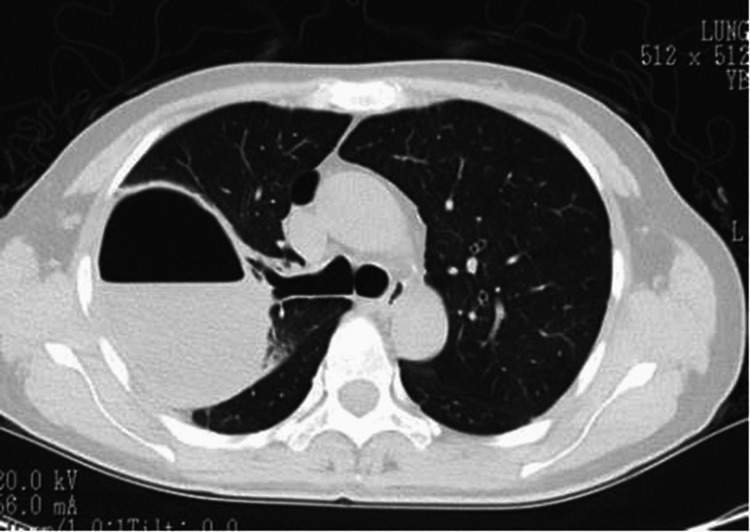
Axial CT showing progression to abscess A thick-walled cavity with pericystic infiltration and fluid level in the right upper lobe is seen, consistent with an abscess.

Despite antibiotics, the patient remained febrile. On day 6, chest X-ray revealed a new air-fluid level (Figure 2), and repeat axial CT showed pericystic infiltration and cyst wall thickening consistent with abscess formation (Figure 3). Due to poor clinical response and concern for sepsis, surgical intervention was planned.

Video-assisted thoracoscopic right upper lobectomy was performed on day 7. Intraoperatively, the cyst contained 150 mL of purulent fluid. Histology confirmed a benign epithelial-lined cyst with neutrophilic infiltration. No neoplasia or parasitic structures were seen, and culture revealed *Streptococcus anginosus*.

The postoperative course was uneventful, with inflammatory markers normalizing (WBC 6,100/μL; CRP 0.5 mg/dL) by day 7 postoperatively. A follow-up chest radiograph was clear. The patient was discharged on day 9 on a seven-day course of oral amoxicillin-clavulanate (1,000 mg/62.5 mg twice daily). No recurrence was observed at the two-month follow-up.

## Discussion

Giant pulmonary cysts (>5 cm) are rare and typically asymptomatic. Complications such as rupture into the pleural space leading to pneumothorax, hemoptysis, secondary infection, and, in rare instances, malignant transformation have been reported [1]. When surgical management is warranted, minimally invasive approaches such as video-assisted thoracic surgery (VATS) have an established safety and efficacy profile in lung cancer surgery [6]. Infectious etiologies must be considered, including bacterial, fungal, and parasitic infections. Hydatid cysts, although rare in Japan, remain important differentials due to global travel and migration [7]. In this case, no evidence supported echinococcal or tuberculous infection. Bronchoscopy may assist in the diagnosis of endobronchial obstruction or neoplasia, but carries risk in cystic lesions, and was not indicated in this case.

*Streptococcus anginosus*, part of the *S. milleri* group, is associated with abscesses in the liver, brain, and lungs [8]. Its isolation justified surgical resection, especially given the poor response to antibiotics. Minimally invasive lobectomy via VATS is a safe approach, even in infectious settings [6]. The lack of sepsis signs, stable vitals, and absence of organ dysfunction do not negate the preemptive value of lobectomy in preventing systemic spread.

## Conclusions

This case illustrates the rare transformation of a pulmonary cyst into a lung abscess, requiring lobectomy despite antibiotic treatment. In such cases, early recognition, monitoring via imaging, and prompt surgical referral are essential to avoid complications. Thoracic surgeons should maintain a broad differential, including hydatid disease, and consider bronchoscopy selectively.
